# Food sources, energy and nutrient intakes of adults: 2013 Philippines National Nutrition Survey

**DOI:** 10.1186/s12937-019-0481-z

**Published:** 2019-10-10

**Authors:** Imelda Angeles-Agdeppa, Ye Sun, Liya Denney, Keith V. Tanda, Royce Ann D. Octavio, Alicia Carriquiry, Mario V. Capanzana

**Affiliations:** 1 Department of Science and Technology, Food and Nutrition Research Institute, Bicutan, Taguig, Philippines; 2 Nestlé Research, Singapore, Singapore; 3 Nestlé Research, Lausanne, Switzerland; 4 Iowa State University, Ames, USA

**Keywords:** Usual nutrient intake, Food sources, Adults, Older adults, The Philippines

## Abstract

**Background:**

Comprehensive assessment of dietary intakes of foods and nutrients in Filipino adults are lacking. This study evaluated energy and nutrient intakes and food sources of key nutrients consumed by Filipino adults.

**Methods:**

The participants were from the 2013 National Nutrition Survey wherein food intake of young adults aged 19–49 years (*n* = 12,896) and older adults aged 50 years and above (*n* = 7853) were collected using 24-h recalls. Usual nutrient intakes were estimated using PC-SIDE program. The Philippines Dietary Reference Intakes were used to calculate proportions of inadequate intake using Estimated Average Requirement (EAR) and Acceptable Macronutrient Distribution Ranges (AMDR). Energy adequacy was evaluated using the Institute of Medicine (IOM) equation for Estimated Energy Requirements (EER).

**Results:**

The nutrient intakes with the highest prevalence of inadequacy (> 50%) were: iron (97–99%), vitamin C (96–98%), calcium (95–98%), riboflavin (86–91%), folate (89–90%), thiamine (73–89%), energy (67–70%), total fat (55–67%), and vitamin A (54–56%). Refined rice, pork and breads contributed most to daily intakes of energy, protein, carbohydrates, thiamine, riboflavin, and iron. Low intake of vegetables, fruits and dairy was common in both age groups.

**Conclusions:**

This study demonstrated that intakes of many nutrients were markedly inadequate among adults in the Philippines, due to the rice-dominant dietary pattern with few nutrient-dense foods. These results can be used to support the development of specific interventions to improve the shortfalls in nutrient intakes.

**Electronic supplementary material:**

The online version of this article (10.1186/s12937-019-0481-z) contains supplementary material, which is available to authorized users.

## Background

Suboptimal diet is associated with a range of non-communicable diseases (NCD), and it is potentially a major contributor to NCD mortality worldwide [1]. In the Philippines, dietary risk is the top risk factor for diseases and is estimated to account for 10.6% of total disability-adjusted life-years [2]. At present, the Philippines hosts the world’s 12th largest population of about 100 million people, among which 7.3% are aged above 60 years. This percentage is expected to double by 2050 with a predicted increase in life expectancy and decrease in fertility rate [3]. However, despite being one of the fastest growing economies in Asia, one out of 10 adults suffers from chronic energy deficiency, and a high proportion (70–80%) of adults is not meeting dietary requirements for many key micronutrients [4–6]. In addition, the prevalence of anemia among older adults is 24% [7]. On the other hand, similar to what happened in many other developing countries in Asia, Africa, and Latin America [8, 9], the Philippines is also experiencing double burden of malnutrition. Three out of 10 adults are overweight or obese [4], and the number of mortality and disability caused by ischemic heart disease, stroke, diabetes and chronic kidney disease has increased by more than 25% in the past decade [10].

The aforementioned nutritional issues increase one’s susceptibility particularly among the elderly to various diseases such as fractures, muscle loss, poorer immunity against infections, and other NCDs [11, 12]. Nutrition-based intervention strategies are one of the key solutions to improve the health status and quality of life of adult population in the Philippines.

In addition, dietary choices could differ within a population under multiple influences, such as age, gender and social economic status (SES). For instance, younger adults might embrace the nutrition transition towards a more “westernized” diet more than the older adults; and gender has an important impact on the social and biological determinants of health consequences, hence different nutritional needs [13]. Identifying such needs and differences is crucial to construct nutritional guidelines and solutions that are tailored to different population groups.

The Food and Nutrition Research Institute (FNRI) in the Philippines conducts National Nutrition Surveys (NNS) every 5 years, which are nationally representative and capture the food and beverage consumption of the Filipino population. However, the existing food composition table (FCT) developed in 1997 only included 12 nutrients, thus limiting the nutrient intakes data being reported. In addition, studies on nutrient intakes of Filipino adults by other researchers are limited both in terms of nutrients coverage and population representativeness. In an attempt to comprehensively characterize the diet of Filipino adults, this study expanded the existing FCT from 12 to 27 nutrients, with which we evaluated the usual intakes of energy and nutrients of adults using data from the 2013 NNS. In addition, the influences of age, gender and SES on nutrient intakes and food sources of key nutrients among adults were also investigated in this study.

## Methods

### Study design and population

This study used the data from the 2013 NNS. This is a cross-sectional, population-based survey that characterizes the health and nutritional status, foods consumption and dietary patterns of the Filipino population. The survey used a multi-staged stratified sampling design to represent all 80 provinces of the country covering both urban and rural areas. A total of 8592 sample households were selected from the NNS for the dietary survey with a response rate of 87.7%. Briefly, 20,749 adults were used in this study, comprising specifically 12,896 aged 19–49 years old representing young adults and 7853 aged 50 years and over representing older adults. The age groups are aligned with the Philippine Dietary Reference Intake age grouping (PDRI, 2015). The Ethics Committee of FNRI approved the survey protocol. All surveyed households provided written informed consent prior to participation.

### Data collection

#### Demographic and socio-economic data

Demographic and socio-economic information were collected from the 2013 NNS survey participants, including age, gender, and area of residence, marital status, education, and the body mass index (BMI). Wealth status of participants was defined by proxy indicators including household possession of vehicles, appliances, materials used for housing construction and sanitation facilities. Scores obtained from principal component analysis were used to define wealth quintiles as poorest, poor, middle, rich and richest. Chronic energy deficiency, overweight and obesity were determined using World Health Organization (WHO) definition [14].

#### Dietary data

Twenty four hours dietary recalls were conducted by registered nutritionist-dietitians through face-to-face interviews in households using structured questionnaires. The interviewer recorded all foods and beverages consumed on the previous day from the moment when they woke up until they went to sleep in the evening. The amount of foods and beverages consumed was estimated using household measures (cups, tablespoons and pieces) or through weighting of food samples. The weights of foods were converted to *as purchased* values using a portion to weight list for common foods compiled by FNRI. If the food was a dish, the interviewee was asked to describe the ingredients of the recipe or name the dish or recipe. The nutrient content of these composite foods were determined by breaking down the different ingredients in the recipe and each was calculated based on INFOODS Guidelines.

A first 24 h recall was collected in all members of all sampled households; and to estimate the day-to-day within-person variability in energy and nutrient intake, a second 24 h recall was carried out among members in 50% of randomly selected households. The repeated 24 h recalls were obtained on non-consecutive days to avoid correlation in nutrient intakes on consecutive days [15]. The values for the two 24 h recalls were averaged for each person to derive their usual intakes. For the remaining 50% of the respondents with only one 24 h recall, their 1 day recall data were unbiased estimate of their usual intake assuming the measurement error is additive [16].

### Data processing

The estimation of energy and nutrients contents of foods consumed was done through the FNRI-Individual Dietary Evaluation System (IDES) which contains the expanded FCT developed from this project. The FCT was expanded from the original 12 nutrients to 27 nutrients, and it is the first time that these 27 nutrients were analyzed in a nationally representative Filipino population. Details about the development of the expanded FCT will be reported in another paper.

Implausible values of energy and nutrient intakes were identified by a process described below. For the evaluation of energy intake, Estimated Energy Requirement (EER) was calculated for each individual using the Institute of Medicine (IOM) equation [17] considering age, sex, body weight, height, and physical activity level (PAL) using the WHO STEP instrument [18]. The ratio of self-reported daily energy intake to the EER was then calculated for each person and each day of reporting. The calculated ratios were then transformed to the logarithmic scale and outliers below and above 3 SDs away from the mean were excluded [19]. Five hundred fifteen subjects were excluded from this exercise. For micronutrients, excessive intakes were defined as those that exceeded 1.5 times of the 99th percentile of the observed intake distribution in the respective age group. Intakes above this upper limit were substituted by a random value generated from a uniform distribution in the interval with lower bound equal to the 95th percentile of observed intake and an upper bound equal to 1.5 times of the 99th percentile [19].

To investigate the food sources of energy and nutrients, a list of 87 food groups under 9 major categories (Table 1) was created in a similar format to the food categories published by United Nations Food and Agriculture Organization (FAO) [20] and United States Department of Agriculture (USDA) [21], while reflecting Filipinos’ frequently consumed foods and traditional way of consumption. All foods, including those less consumed foods, were considered in the analysis.

**Table 1:** Food group classification

Milk	Vegetables	Sweets
Adult formula (fortified milk powder)	Dark green leafy vegetables	Sweet bakery products
Cow’s milk (fluid and powdered)	* Spinach*	* Cookies*
Other milk	* Broccoli*	* Biscuits*
Cheese	* Cabbage, green*	* Sweet breads*
Yoghurt	* Local leafy/petioles/salad vegetables*	* Cakes*
Meats/Fish/other protien sources	Deep yellow vegetables	Ice cream, popsicles
Beef	* Carrot*	Candy
Carabeef	* Sweet potato, yellow*	Sugar
Pork	* Cassava, yellow*	Syrup
Goat/lamb	* Squash fruit*	Preserves/jams/jellies
Chicken	* Squash, summer fruits*	Native desserts/snacks
Duck	Starchy vegetables	Sugar sweetened beverages
Sausages/hotdogs	* Sweet potato*	* Fruit-based beverages*
Luncheon meats/cold cuts	* Potato*	* Concentrated fruit juice drinks*
Fish	Other vegetables	* Powdered fruit flavored drinks*
Eggs	Vegetable products/processed vegetables	* Soft drinks*
Beans/nuts	Fruit & 100% fruit juice	* Chocolate/chocolate flavor beverages*
Grains & Grain products	Apple	* Other sweetened beverages*
Refined rice	Avocado	Mixed dishes
Cereal	Banana	Meat-based mixed dishes
Bread	Mango	Beans-based mixed dishes
Crackers	Melon	Grain-based mixed dishes
Pancakes, waffles, French toast	Citrus fruits	Soups
Noodles	Cherries/berries	Other foods & beverages
Pasta	Papaya	Non-alcoholic beverages
Corn grits	100% Fruit juice	Alcoholic beverages
Cornmeal	fats/Oils	Savory snacks
	Fats	Condiments, sauces, herbs, spices, other seasonings
	Oils	

### Statistical analysis

Mean and usual intake distributions of energy and nutrients were estimated using the PC-SIDE software (Software for Intake Distribution Estimation version 1.0, Iowa State University, IA, USA) [22]. This method developed by Iowa State University could account for the within-person variability of daily intakes across different days, and therefore only reflecting the between-person variability [16]. To determine if the mean differences of usual nutrient intakes across different age and gender subgroups were statistically significant, Analysis of Covariance (ANCOVA) was used with adjustment for total energy intake.

PDRI was used to evaluate nutrient inadequacies [23]. Where applicable, the prevalence of inadequacy in a group is estimated as the proportion of individuals with usual intakes below the Estimated Average Requirement (EAR), using the EAR cut-point method [24]. Due to a skewed distribution of iron intake, a probability approach was used instead to assess the prevalence of inadequate iron intake: the risk of inadequacy of each individual was computed first, and the prevalence of inadequate intake was estimated as the average risk of inadequacy [25]. Intakes of carbohydrates, fat, and protein were evaluated as percentage of total energy intake, and inadequacy or excessive intake was classified as less than the lower limit or higher than the upper limit of the Acceptable Macronutrient Distribution Ranges (AMDR). Additional file 1: Table S1 summarizes the EAR and AMDR benchmarks used in this study. Assessment of nutrient adequacy was also computed by gender, age groups, and wealth quintiles, and hypothesis testing comparing two population proportions was used to test the differences in prevalence of inadequacies across various subgroups.

Stata (Stata Statistical Software: Release 15. StataCorp, TX, USA) was used for data management, calculation of summary statistics, and statistical tests of differences. A *p*-value of < 0.05 was considered significant in all statistical tests. Survey weights were applied in all datasets and calculations to represent national estimates through the complex survey design.

## Results

### Demographic and socio-economic characteristics, and nutritional status of the study population

Table 2 summarizes the demographic and socio-economic characteristics of the two age groups. Among the young adults and the older adults, respectively 53.8 and 45.1% were males. Approximately half of the study population resided in urban areas, and they were approximately equally distributed across the 5 wealth quintiles. Half of the older adults (51.0%) only attained elementary education, while majority of the younger adults completed high school or higher education (74.8%). The prevalence of chronic energy deficiency among young and older adults was 10.4 and 15.5% respectively, while 27.7 and 28.5% were overweight/obese.

**Table 2:** Demographic, socio-economic characteristics and nutritional status of the study population

	19–49 years		50 years and above	
	*n*	%	*n*	%
Total, n	12,896		7853	
Age (mean ± SE)	34.4 ± 0.08		62.6 ± 0.1	
Gender				
Male	6935	53.8	3539	45.1
Female	5961	46.2	4314	54.9
Area of residence				
Urban	6907	53.6	4464	56.8
Rural	5989	46.4	3389	43.2
Wealth quintiles				
Poorest	2557	20.3	1542	20.1
Poor	2653	21.1	1638	21.4
Middle	2594	20.6	1552	20.3
Rich	2454	19.5	1453	19.0
Richest	2308	18.4	1468	19.2
Marital status				
Single	4331	33.6	431	5.5
Married	6728	52.2	5149	65.6
Live-in	1351	10.5	327	4.2
Widow	177	1.4	1776	22.6
Separated / divorced	304	2.4	167	2.1
Educational attainment				
No grade completed	196	1.5	369	4.7
Elementary level	3023	23.6	3986	51.0
High school level	5324	41.5	2062	26.4
College level	3386	26.4	1168	14.9
Vocational level	887	6.9	226	2.9
Others	17	0.1	6	0.1
Body mass index (kg/m^2^)				
≤ 18.5 (chronic energy deficiency)	1288	10.4	1149	15.5
18.5 to 24.99 (normal)	7652	61.8	4137	56.0
≥ 25.0 (overweight / obese)	3432	27.7	2105	28.5

### Energy and macronutrient intakes

Table 3 summarizes the mean usual intakes of energy and nutrients by age and gender subgroups. The mean usual energy intake (mean ± standard error) was 1828 ± 6 kcal/day (young adults) and 1527 ± 6 kcal/day (older adults), which was 30.2 and 33.5% lower than the mean estimated EER of 2620 ± 4 kcal/day and 2297 ± 5 kcal/day respectively.

**Table 3:** Mean usual nutrient intakes of Filipino adults by age and gender groups: 8th NNS 2013

Nutrients	19–49 years (young adults)			50 years and above (older adults)		
	Both genders	Gender		Both genders	Gender	
		Male	Female		Male	Female
Sample, *n*	12,896	6935	5961	7853	3593	4314
Macronutrients						
Energy (kcal/d)	1828 ± 6^a^	2096 ± 8^b^	1535 ± 6	1527 ± 6	1767 ± 7^b^	1322 ± 6
Total fat (g/d)	32.2 ± 0.2^a^	34.7 ± 0.3^b^	29.5 ± 0.2	21.2 ± 0.2	23.2 ± 0.4^b^	19.6 ± 0.2
Saturated fat (g/d)	14.3 ± 0.1^a^	15.2 ± 0.1 ^**NS**^	13.4 ± 0.1	10.3 ± 0.1	11.0 ± 0.1 ^**NS**^	10.0 ± 0.1
MUFA (g/d)	10.8 ± 0.1^a^	11.6 ± 0.1^b^	10.1 ± 0.1	7.5 ± 0.1	8.0 ± 0.1 ^**NS**^	7.0 ± 0.1
PUFA (g/d)	5.3 ± 0.1^a^	5.9 ± 0.1 ^**NS**^	4.8 ± 0.1	3.7 ± 0.1	3.9 ± 0.1^b^	3.4 ± 0.1
Protein (g/d)	60.1 ± 0.2^a^	68.7 ± 0.3 ^**NS**^	50.7 ± 0.2	50.1 ± 0.2	58.3 ± 0.3^b^	43.0 ± 0.2
Carbohydrates (g/d)	322 ± 1^**NS**^	372 ± 1^b^	266 ± 1	273 ± 1	317 ± 2 ^**NS**^	237 ± 1
Total sugars (g/d)	26.3 ± 0.1^a^	25.0 ± 0.2 ^**NS**^	28.0 ± 0.2	25.7 ± 0.2	24.7 ± 0.2 ^**NS**^	26.5 ± 0.2
Dietary fiber (g/d)	9.1 ± 0.03^a^	10.1 ± 0.1^b^	8.0 ± 0.04	8.5 ± 0.04	9.5 ± 0.1^b^	7.6 ± 0.1
As percentage of total energy						
Total fat (%)	15.1 ± 0.1^a^	14.0 ± 0.5^b^	16.3 ± 0.1	13.2 ± 0.1	12.4 ± 0.1 ^**NS**^	13.8 ± 0.1
Protein (%)	13.3 ± 0.02^a^	13.2 ± 0.04^b^	13.4 ± 0.02	13.4 ± 0.02	13.5 ± 0.04^b^	13.3 ± 0.03
Carbohydrates (%)	71.1 ± 0.1^a^	71.9 ± 0.1^b^	70.3 ± 0.1	73.0 ± 0.1	73.2 ± 0.1 ^**NS**^	72.9 ± 0.1
Vitamins						
Thiamine (mg/d)	0.8 ± 0.01^a^	0.9 ± 0.01^b^	0.7 ± 0.01	0.6 ± 0.03	0.7 ± 0.01^b^	0.6 ± 0.03
Riboflavin (mg/d)	0.7 ± 0.01^a^	0.8 ± 0.01 ^**NS**^	0.6 ± 0.01	0.6 ± 0.04	0.6 ± 0.01^b^	0.5 ± 0.03
Niacin (mg NE/d)	19.5 ± 0.1^a^	22.3 ± 0.1 ^**NS**^	16.4 ± 0.1	16.6 ± 0.1	19.2 ± 0.1^b^	14.2 ± 0.1
Vitamin B6 (mg/d)	1.6 ± 0.01^a^	1.8 ± 0.01^b^	1.5 ± 0.01	1.4 ± 0.01	1.5 ± 0.01^b^	1.2 ± 0.01
Vitamin B12 (μg/d)	4.0 ± 0.01^a^	4.4 ± 0.02^b^	3.5 ± 0.02	3.6 ± 0.02	4.2 ± 0.03^b^	3.1 ± 0.02
Vitamin A (μg RE/d)	470 ± 2^a^	505 ± 3^b^	433 ± 3	408 ± 3	447 ± 4^b^	375 ± 3
Vitamin C (mg/d)	23.4 ± 0.1^a^	22.7 ± 0.2^**NS**^	22.8 ± 0.2	24.1 ± 0.2	24.4 ± 0.3 ^**NS**^	23.8 ± 0.2
Vitamin E (mg α-TE/d)	2.9 ± 0.01^a^	3.2 ± 0.02^b^	2.6 ± 0.02	2.5 ± 0.02	2.8 ± 0.03^b^	2.3 ± 0.02
Vitamin D (μg/d)	3.2 ± 0.01^a^	3.5 ± 0.02^b^	2.9 ± 0.02	3.0 ± 0.02	3.4 ± 0.03^b^	2.7 ± 0.02
Folate (μg DFE/d)	190 ± 1^a^	203 ± 1^b^	177 ± 1	182 ± 1	196 ± 2^b^	171 ± 2
Minerals						
Calcium (mg/d)	312 ± 1^a^	340 ± 1^b^	281 ± 1	308 ± 2	328 ± 2^b^	268 ± 2
Iron (mg/d)	8.8 ± 0.03^a^	9.6 ± 0.04^b^	7.9 ± 0.04	7.5 ± 0.04	8.2 ± 0.1^b^	6.7 ± 0.04
Phosphorus (mg/d)	882 ± 3^a^	1012 ± 4 ^**NS**^	739 ± 3	750 ± 3	869 ± 5 ^**NS**^	647 ± 3
Sodium (mg/d)	821 ± 3^a^	851 ± 5^b^	817 ± 5	727 ± 5	699 ± 6^b^	667 ± 5
Zinc (mg/d)	6.8 ± 0.1^a^	7.5 ± 0.1 ^**NS**^	5.8 ± 0.1	5.5 ± 0.1	6.2 ± 0.1 ^**NS**^	4.9 ± 0.1
Magnesium (mg/d)	181 ± 1^a^	204 ± 1^b^	155 ± 1	159 ± 1	182 ± 1^b^	140 ± 1
Potassium (mg/d)	1271 ± 4^a^	1392 ± 5^b^	1140 ± 5	1155 ± 5	1282 ± 8^b^	1046 ± 6
Selenium (μg/d)	103.1 ± 0.3^a^	117.8 ± 0.5^b^	87.3 ± 0.4	84.5 ± 0.4	97.0 ± 0.6^b^	73.6 ± 0.4

*Abbreviations*: *MUFA* Monounsaturated fatty acids, *PUFA* Polyunsaturated fatty acids, *NE* Niacin equivalent, *RE* Retinol equivalent, *α-TE* α-tocopherol equivalent, *DFE* Dietary folate equivalent

Values shown are mean ± standard error of usual nutrient intakes

^a^Statistically significant difference between young adults and older adults with adjustment for total energy intake by ANCOVA test. ^NS^ Not significant

^b^Statistically significant difference between male and female adults of the same age group with adjustment for total energy intake by ANCOVA test. ^NS^ Not significant

Overall, younger adults consumed significantly more energy and most of the macronutrients than the older adults with the exception of carbohydrates. Males consumed significantly higher energy and many nutrients than females within both age groups. It is also worth noting that the mean consumption of dietary fiber, ranged from 7.6–10.1 g, is far below the recommended nutrient intake of 20–25 g/day for adults.

When examined as percentage of total energy, fat, protein, and carbohydrates contributed to 12.4–16.3%, 13.2–13.5%, and 70.3–73.2% of daily energy intake, respectively. Comparing against the AMDR recommendations, 55–67% of the study population did not consume adequate fat (Table 4). The prevalence of inadequate fat intake was significantly higher in older adults, among males (Table 4), and in poor and poorest wealth quintiles (Table 5).

**Table 4:** Prevalence of inadequacies of usual nutrient intakes among Filipino adults by age and gender groups^a^

Nutrients	19–49 years (young adults)			50 years and above (older adults)		
	Both genders	Gender		Both genders	Gender	
		Male	Female		Male	Female
Sample, *n*	12,896	6935	5961	7853	3593	4314
Total fat (%) ^b^	55†	62^*^	48	67	72^*^	63
Protein (%) ^b^	2^**NS**^	2 ^**NS**^	2	3	3 ^**NS**^	3
Carbohydrates (%) ^b^	3 ^**NS**^	3 ^**NS**^	3	2	2 ^**NS**^	1
Protein (g/d)	42†	33^*^	51	62	53^*^	70
Thiamine (mg/d)	73†	68^*^	81	89	86^*^	92
Riboflavin (mg/d)	86†	87^**NS**^	86	91	94 ^**NS**^	94
Niacin (mg NE/d)	10†	5^*^	13	22	14^*^	28
Vitamin A (μg RE/d)	54†	53^*^	55	66	65^*^	67
Vitamin C (mg/d)	98†	99^*^	97	96	97^*^	95
Vitamin B6 (mg/d)	28†	17^*^	38	61	55^*^	64
Vitamin B12 (μg/d)	9†	6^*^	13	17	11^*^	23
Folate (μg DFE/d)	90†	86^*^	93	89	86^*^	92
Iron (mg/d)	97†	95^*^	99	99	95^*^	97
Calcium (mg/d)	98†	97^*^	99	95	95^*^	99
Phosphorus (mg/d)	14†	6^*^	24	30	16^*^	42
Zinc (mg/d)	14†	15^*^	10	34	34^*^	27
Selenium (μg/d)	< 1^**NS**^	< 1^**NS**^	< 1	1	1^NS^	1

*Abbreviations*: *NE* Niacin equivalent, *RE* Retinol equivalent, *DFE* Dietary folate equivalent

^a^8th NNS 2013

Values shown are percentages of study sample below daily Estimated Average Requirement (EAR), with the exception of ^b^ Acceptable Macronutrient Distribution Range (AMDR)

^†^Statistically significant difference between young adults and older adults, *P* < 0.05. ^NS^ Not significant

^*^Statistically significant difference between male and female adults of the same age group, *P* < 0.05. ^NS^ Not significant

**Table 5:** Prevalence of inadequacies of usual nutrient intakes among Filipino adults by age and status^a^

Nutrients	19–49 years (young adults)					50 years and above (older adults)				
	Wealth quintiles					Wealth quintiles				
	Poorest	Poor	Middle	Rich	Richest	Poorest	Poor	Middle	Rich	Richest
Sample, *n*	2577	2653	2594	2454	2308	1542	1638	1552	1453	1468
Total fat (%) ^b^	90^*^	77	64	37	17	91^*^	86	76	55	33
Protein (%) ^b^	7^*^	3	1	< 1	< 1	9^*^	4	2	< 1	1
Carbohydrates (%) ^b^	< 1^*^	< 1	< 1	3	9	< 1^*^	< 1	< 1	1	3
Protein (g/d)	57^*^	50	41	32	30	74^*^	70	64	57	48
Thiamine (mg/d)	86^*^	81	75	66	60	91^*^	91	89	85	80
Riboflavin (mg/d)	94^*^	92	89	81	76	95^*^	94	93	91	85
Niacin (mg NE/d)	22^*^	15	8	4	5	36^*^	29	19	17	12
Vitamin A (μg RE/d)	56^*^	68	59	50	46	70^*^	74	72	70	62
Vitamin C (mg/d)	96^*^	98	99	98	98	89^**NS**^	91	97	95	89
Vitamin B6 (mg/d)	40^*^	38	28	21	14	70^*^	66	65	61	45
Vitamin B12 (μg/d)	14^*^	13	10	4	4	27^*^	22	14	9	8
Folate (μg DFE/d)	84^*^	88	93	91	93	83^*^	88	92	94	91
Iron (mg/d)	99^*^	98	98	96	95	97^*^	95	95	93	87
Calcium (mg/d)	98^*^	98	98	98	96	95^*^	94	95	96	97
Phosphorus (mg/d)	23^*^	17	13	10	11	40^*^	35	27	28	20
Zinc (mg/d)	29^*^	21	16	5	4	53^*^	43	33	25	15
Selenium (μg/d)	1^**NS**^	1	< 1	0	0	4^*^	2	1	< 1	0

*Abbreviations*: *NE* Niacin equivalent, *RE* Retinol equivalent, *DFE* Dietary folate equivalent

^a^8th NNS 2013

Values shown are percentages of study sample below daily Estimated Average Requirement (EAR), with the exception of ^b^ Acceptable

Macronutrient Distribution Range (AMDR)

^*^Statistically significant difference between poorest and richest adults of the same age group, *P* < 0.05. ^NS^ Not significant

Protein intake was also evaluated with the EAR in g/day. Unlike when comparing with AMDR, a high prevalence of inadequacy was observed across all age and gender groups, with a more serious situation for older adults, females (Table 4), and in poor and poorest wealth quintiles (Table 5).

### Micronutrient intakes

High prevalence of inadequate micronutrient intakes were found for iron (97–99%), vitamin C (96–98%), calcium (95–98%), folate (89–90%), riboflavin (86–91%), thiamine (73–89%), and vitamin A (54–66%) (Table 4). For micronutrients with no established EAR recommendations, including vitamin D, vitamin E, magnesium and potassium, the mean intakes were also far from the adequate intakes.

On average, mean usual intakes of most vitamins and minerals were siginificantly higher in young adults than in older adults. A differing result was observed for vitamin C as the average intake of older adults is higher than that of younger adults, though both were far below the EAsR of 52–60 mg/d. In both age groups, the mean consumption of male adults for most vitamins and minerals was significantly higher than females (Table 3).

Corresponding to the differences observed in mean usual intakes, the prevalence of inadequacy increases significantly with age for many micronutrients, in particular thiamine, niacin, vitamin A, vitamin B6, vitamin B12, phosphorus, and zinc. Also, females might be at higher risk of inadequacy for thiamine, niacin, vitamin A, vitamin B6, vitamin B12, folate, iron, calcium and phosphorus than males in both age groups, while males might be at higher risk for vitamin C and zinc inadequacy (Table 4).

Higher prevalence of inadequacy was observed among the poorest group for most micronutrients. It is worth noting that the prevalence of inadequacy in vitamin C, folate, iron and calcium remained high across the wealth quintiles, and that more than 50% of adults did not consume adequate vitamin C, folate, riboflavin, thiamine, vitamin A (only for older adults), iron and calcium even in the highest wealth index group (Table 5).

### Consumption rate and mean consumption per capita of major food groups

Grains, meat and proteins, sweets and vegetables were the top 4 major food groups consumed in both age groups in terms of consumption rate as well as mean intake per capita (Table 6). Grains, mainly refined rice, played a dominant role in Filipino’s diet (mean per capita 290.8–350.4 g/day). Only less than 25% of adults consumed fruit, and even fewer consumed milk (9.3–13.4%). The mean consumption per capita of vegetables (66.4–70.1 g/day), fruit (24.4–29.7 g/day) and milk (2.8–3.2 g/day) was far from the recommended 3 servings/day of vegetables, 2–3 servings/day of fruit, and 1 glass/day of milk [26], and this could explain partially the high prevalence of nutrient inadequacies.

**Table 6:** Population consumption rate and mean consumption per capita of major food groups among Filipino adults^a^

19–49 years (young adults)				50 years and above (older adults)			
Rank	Food groups	% of adults consuming	Mean intake (SE) per capita (g)	Rank	Food groups	% of adults consuming	Mean intake (SE) per capita (g)
1	Grains	99.5	350.4 ± 0.6	1	Grains	99.6	290.8 ± 0.6
2	Meat & proteins	94.8	152.3 ± 0.5	2	Meat & proteins	92.7	117.4 ± 0.5
3	Sweets	76.9	102.3 ± 0.9	3	Sweets	79.2	63.4 ± 0.8
4	Vegetables	68.5	66.4 ± 0.4	4	Vegetables	68.9	70.1 ± 0.5
5	Fats & oils	63.1	6.2 ± 0.1	5	Other foods & beverages	64.6	25.7 ± 1.4
6	Other foods & beverages	62.7	35.9 ± 1.4	6	Fats & oils	57.4	5.1 ± 0.1
7	Fruit	21.2	24.4 ± 0.8	7	Fruit	24.9	29.7 ± 0.9
8	Milk	9.3	2.8 ± 0.3	8	Milk	13.4	3.2 ± 0.2
9	Mixed dishes	1.9	5.1 ± 1.5	9	Mixed dishes	2.1	6.0 ± 1.4

^a^8th NNS 2013

### Food sources of energy and nutrients

**Fig. 1:**
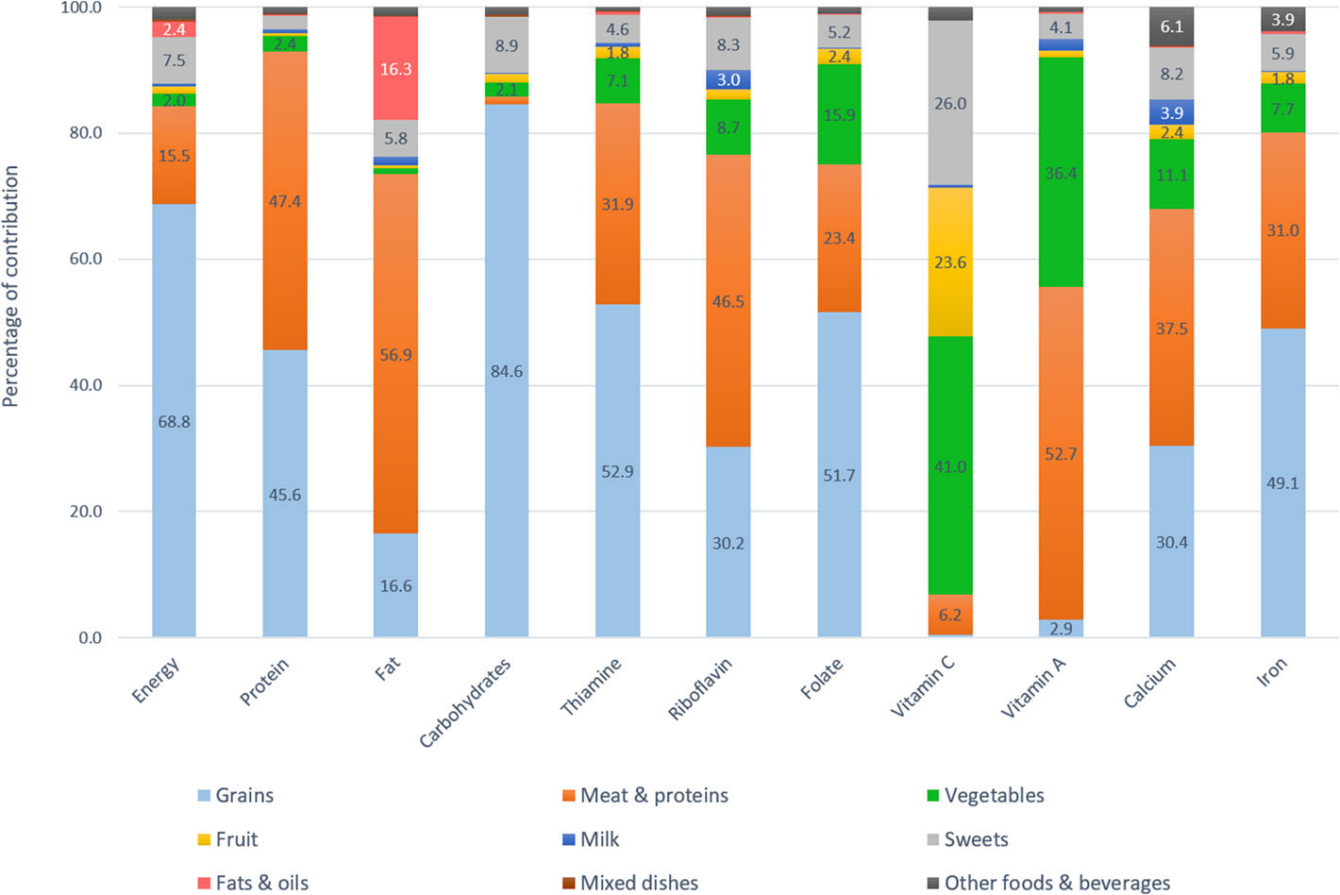
Food sources of energy and key nutrients among adults aged 19 to 49 years

**Fig. 2:**
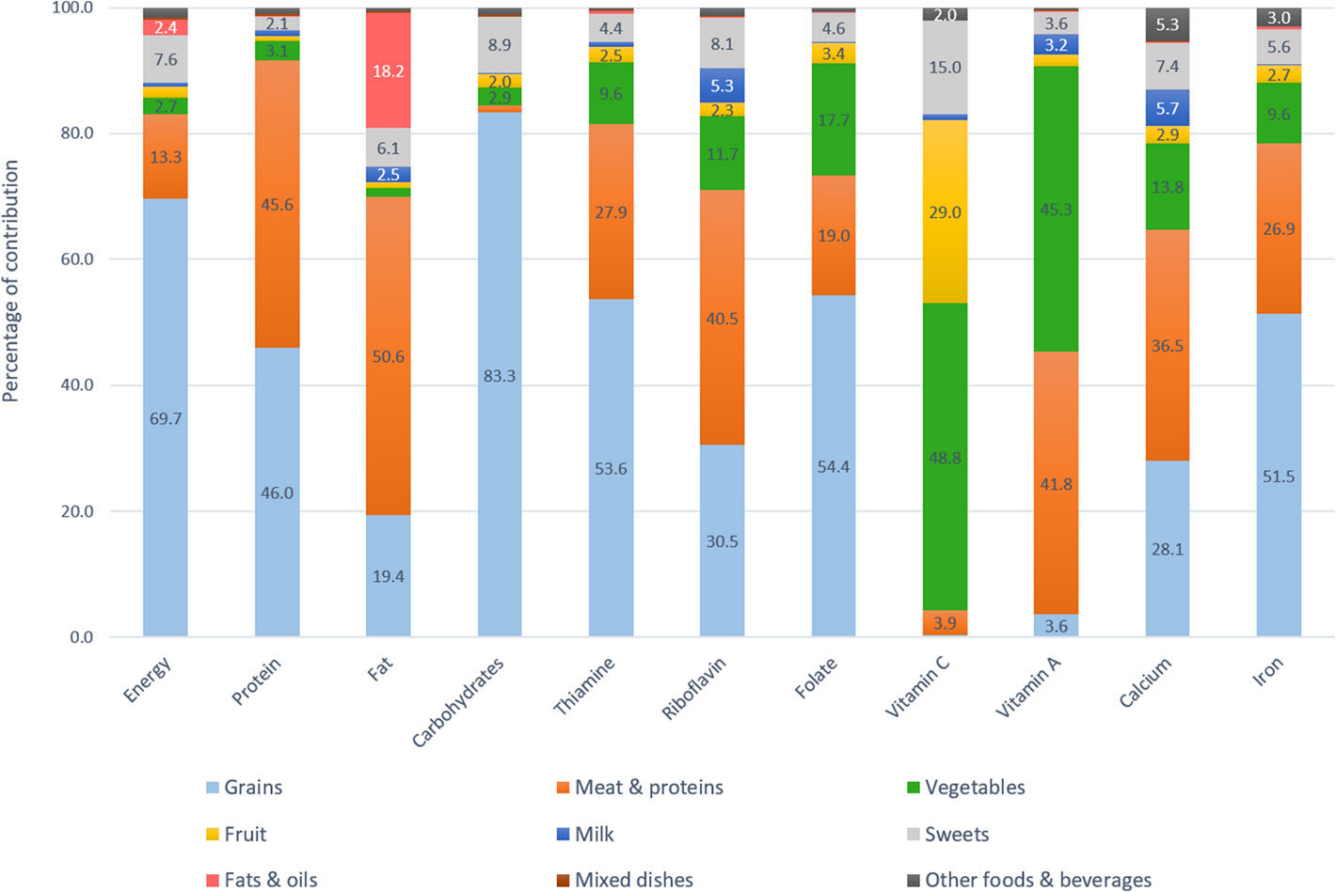
Food sources of energy and key nutrients among older adults aged 50 years and above

Figures 1 and 2 depict percentage contribution of the 9 major food groups to energy, macronutrients, and micronutrients with high prevalence of inadequacy (> 50%). The top 3 major food sources of energy were grains (68.8–69.7%), meats and other protein-rich foods (13.3–15.5%), and sweets (7.5–7.6%).

Grains contributed nearly 70% of daily energy, more than 80% of carbohydrates, half of thiamine, folate, iron, and protein, and one third of calcium and riboflavin. Meat and other protein-rich foods were the source of half of fat, protein, vitamin A, and riboflavin intakes, one third of thiamine, calcium, and iron intakes, and one fifth of folate intake. Vegetables contributed 40–50% of vitamin C, 40% of vitamin A, and less than 20% of folate, calcium, and 10% of riboflavin, thiamine, and iron. Approximately 20–30% of vitamin C was from fruits and a similar percentage from sweet foods, mainly from fruit-based beverages and fortified sweetened beverages. Milk only contributed 0.4–0.7% of total energy intake due to the very low consumption rate in this population, but being a nutrient-dense food, milk still contributed 3.9–5.7% of calcium, 3–5.3% of riboflavin, and 1.9–3.2% of vitamin A. Fats and oils contributed less than 20% of total fat intake, and minimal amounts to other nutrients. Mixed dishes and other foods and beverages played a little role in the energy and nutrient intakes. The top major sources of energy and nutrients are also available (see Additional file 2: Table S2, Additional file 3: Table S3 and Additional file 4: Table S4.

## Discussion

Our study investigated the usual intakes of energy and nutrients, and their primary food sources among young and older adults in the Philippines. To our knowledge, this is the first study to provide a comprehensive overview of the dietary intakes of 27 macro- and micro-nutrients among Filipino adults with a nationally representative population sample. Our findings provided important insights on the sub-optimal dietary patterns of Filipino adults, and as a result, the large shortfalls of intakes in many nutrients.

The mean energy intake of both young and older adults was approximately 30% lower than the recommended intake, which corroborates with the observation, that 10–15% of them suffer from chronic energy deficiency. In addition, a substantial decline with age in daily energy intakes was observed, which is consistent with many other populations [27, 28]. The energy needs decrease as people age, possibly attributable to the decrease in muscle mass, physical activity level, and overall basal metabolic rate [28, 29]. On the other hand, the ageing process could mean reduced ability to absorb and metabolize certain nutrients [30]. It is therefore important for older adults to consume more nutrient-dense foods in order to fulfill their nutritional needs.

The contribution of carbohydrates and protein to energy intake were within the AMDR. However, It is worth noting that the AMDR reference ranges for protein used in Philippines is 10–15% of total energy intake, which is much narrower towards the lower boundary as compared with 10–35% used by the Institute of Medicine [17]. In addition, the fact that about half of the Filipino adults’ daily protein intake was from grains, mainly refined white rice, suggested a poor quality of dietary protein [31]. The development of EAR for protein in PDRI has taken into consideration the protein quality in Filipino rice-based diet [32], and when compared with the protein EAR, 42–62% of adults did not meet the recommendation. On the other hand, the low contribution of fats to energy may pose certain problems on the absorption and utilization of fat-soluble vitamins.

A high prevalence of inadequacy was also observed for many vitamins and minerals crucial for adults’ optimal health: 50–99% of adults did not eat adequate vitamin C, calcium, iron, folate, riboflavin, thiamine, vitamin A, and vitamin B6 (only among older adults), while 25–50% of adults did not meet the EAR for vitamin B6 (young adults), zinc and phosphorus (older adults). In addition, population mean intakes of fiber, vitamin D, vitamin E, magnesium and potassium were far below the adequate intakes. These findings are in general consistent with previous reports in Filipino adults using different dietary intake assessment methods [33–35]. Compared to the previous NNS conducted in 2008, there was little improvement in the nutritional inadequacies [35]. Nutrients all play different, yet pivotal roles in the body, and insufficient intakes could increase one’s susceptibility to various diseases. The inadequacy of blood-forming nutrients such as folate, vitamin B6, vitamin B12, and iron may lead to higher susceptibility to anemia [36–38], increased coronary heart disease risk [39] or poor cognitive outcomes in older adults [40, 41]. Moreover, inadequacy of calcium and vitamin D may increase the risk of osteoporosis and frailty in old age [42, 43]. Older people may be more vulnerable to calcium and vitamin D deficiency due to poorer absorption of calcium, reduced vitamin D synthesis in the skin, and decreased ability of the kidney to convert vitamin D to its active form [44]. The markedly high prevalence of calcium inadequacy (95–98%) in our study population could be explained by the very low intake of dairy products. Many tropical countries still report a considerable proportion of the population having insufficient vitamin D levels due to more time spent indoors and less sunlight exposure [45–47]. Food fortification with vitamin D has been proposed considering that the natural food sources of vitamin D are not commonly consumed in the studied population [43].

The Filipino diet is of limited diversity wherein white rice, pork and breads contributed most to daily intake of energy, protein, carbohydrates, thiamine, riboflavin, and iron. Many nutrient-dense food groups such as vegetables, fruit, and dairy were seriously lacking in the diet. Although vegetables and fruits were the top two food sources for vitamin C and folate, less than 70% of the population consumed vegetables daily, and even fewer (less than 25%) consumed fruit, and the amount of consumption was not sufficient to support adults’ nutrition needs. Dairy foods, with only 0.4–0.7% of energy contribution, were the source of 3.9–5.7% of dietary calcium, 3–5.3% of riboflavin, and 1.9–3.2% of vitamin A. Increasing dairy consumption could improve the dietary intake of these key nutrients.

This study also investigated the nutrient intake status across various population subgroups including age, gender, and SES. In general, the prevalence of inadequacy increases with age for most nutrients, in particular thiamine, niacin, vitamin A, vitamin B6, vitamin B12, phosphorus, and zinc. This is due to not only overall reduced food consumption as people age, but also increased nutritional needs because of poorer absorption and metabolism [30]. Also, in both age groups, females are at higher risk of inadequate for thiamine, niacin, vitamin A, vitamin B6, vitamin B12, folate, iron, calcium and phosphorus than males, while males are at higher risk for vitamin C and zinc inadequacy. Although it has been observed in many developed countries that women were more likely to engage in healthy living and healthy dietary choices [48, 49], studies conducted in developing countries as with our study generally reported better nutritional status among the males than females, likely because of the gender differences in social and economic aspects [15]. Lastly, it was observed that the prevalence of inadequate nutrient intake decreases as wealth status progresses, which was also observed in previous studies [50, 51]. However, increasing SES does not necessarily mean better nutritional status [52]. As demonstrated in the present study, inadequate intake of many key nutrients such as vitamin C, iron, calcium, folate and protein remained high even among the richest wealth quintile. Such inadequacies are likely due to the population-wide dietary pattern with low consumption of nutrient-dense foods including fruit, vegetables, and milk. These results demonstrate that overall nutrient intake and dietary diversity need to be improved, with a special focus on interventions for the elderly, females, and those in low SES and food insecure.

This study has provided a comprehensive summary of the dietary intakes and nutritional status of Filipino adults and older adults. The use of mean intakes provided a general overview of nutrient intake levels of the population, while the EAR cut-point method with the national representative sample allowed an estimate of the prevalence of the population with inadequacy intakes. Detailed segmentation of the studied sample by age, gender and SES is instrumental in constructing future tailored nutritional solutions to meet the needs of specific subgroups of the population. However, our study also has several methodological limitations. Firstly, the use of 24-h recalls to collect dietary intake data relies on the participants’ ability to accurately recall the foods consumed and estimate the portion sizes of consumption. Secondly, information on use of dietary supplements was not captured in this study, which could under-estimate the nutrient intakes. Thirdly, the construction of the Filipino FCT involved matching similar food items with established databases such as USDA, while in reality, the nutritional content could be different for similar foods, due to different breed cultivars, climate conditions, mineral abundance in soil, and national food fortification policies. Therefore, the findings reported in this study could be subject to measurement errors, and it is warranted to, if possible, relate these dietary intake data with nutritional biomarkers and health conditions to facilitate better interpretation.

## Conclusion

Our findings provided important insights to the dietary patterns of Filipino adults, and showed that marked nutrient inadequacies exist in the adult population, especially among older adults, females, and people from lower SES. The lack of dietary variety and nutritional quality could explain the large shortfalls of many nutrient intakes. A large proportion of energy intake was from foods with low nutrient density such as refined rice and sweets. Nutrient-dense foods such as vegetables, fruits, and dairy products being the least nutrient contributors as shown in the study, should be greatly encouraged to fulfill the nutritional gaps. Food fortification targeting nutrients that are commonly inadequate in the population should also be considered. Together, the findings can help to support the development of specific interventions to improve nutritional status especially among those more vulnerable to dietary inadequacies.

## Additional files

Additional file 1: Dietary reference intakes of nutrients for Filipino adults and older adults. (DOCX 16 kb)

Additional file 2: Ranking of foods as major sources of energy, protein, total fat, and carbohydrates among adults (19 years and above). (DOCX 18 kb)

Additional file 3: Ranking of foods as major sources of thiamine, riboflavin, vitamin A, and vitamin C among adults (19 years and above). (DOCX 19 kb)

Additional file 4: Ranking of foods as major sources of folate, calcium, and iron among adults (19 years and above). (DOCX 16 kb)

## Data Availability

All data generated or analysed during this study are included in this published article and its supplementary information files.

## Abbreviations

AMDR: Average Macronutrient Distribution Range
ANCOVA: Analysis of Covariance
BMI: Body Mass Index
DFE: Dietary Folate Equivalent
EAR: Estimated Average Requirement
EER: Estimated Energy Requirements
FAO: Food and Agriculture Organization
FCT: Food Composition Table
FNRI: Food and Nutrition Research Institute
IDES: Individual Dietary Evaluation Systems
IOM: Institute of Medicine
MUFA: Monounsaturated Fatty Acids
NE: Niacin Equivalent
NNS: National Nutrition Survey
PAL: Physical Activity Level
PDRI: Philippine Dietary Reference Intake
PUFA: Polyunsaturated Fatty Acids
RE: Retinol Equivalent
SES: Social Economic Status
USDA: United States Department of Agriculture
WHO: World Health Organization
α-TE: α-tocopherol equivalent
