# Supplementary material for: Food sources, energy and nutrient intakes of adults: 2013 Philippines National Nutrition Survey
Source: Nutr J. 2019 Oct 10;18:59. doi: 10.1186/s12937-019-0481-z (PMC6785859; doi:10.1186/s12937-019-0481-z)
Supplement: Supplementary file 1 — Dietary reference intakes of nutrients for Filipino adults and older adults. (DOCX 16 kb) [file 12937_2019_481_MOESM1_ESM.docx]

**Additional file 1: Table S1.** Dietary reference intakes of nutrients for Filipino adults and older adults^1^

| **Nutrients** | **19-49 years (adults)** | | **50 years and above (older adults)** | |
| --- | --- | --- | --- | --- |
|  | **Male** | **Female** | **Male** | **Female** |
| ***Macronutrients*** |  |  |  |  |
| Protein (g/d) | 57 | 49 | 57 | 49 |
| Fiber (g/d) **^a^** | 20-25 | | 20-25 | |
| ***As percentage of total energy*** |  |  |  |  |
| Total Fat (%) **^b^** | 15-30 | | 15-30 | |
| Protein (%) **^b^** | 10-15 | | 10-15 | |
| Carbohydrates (%) **^b^** | 55-75 | | 55-75 | |
| ***Vitamins*** |  |  |  |  |
| Vitamin A (µg RE/d) | 499 | 433 | 499 | 433 |
| Vitamin C (mg/d) | 60 | 52 | 60 | 52 |
| Thiamine (mg/d) | 1 | 0.9 | 1 | 0.9 |
| Riboflavin (mg/d) | 1.1 | 0.9 | 1.1 | 0.9 |
| Niacin (mg NE/d) | 12 | 11 | 12 | 11 |
| Vitamin B6 (mg/d) | 1.1 | 1.1 | 1.4 | 1.3 |
| Vitamin B12 (µg/d) | 2 | 2 | 2 | 2 |
| Vitamin D (µg/d) ^a^ | 5 | 5 | 15 | 15 |
| Vitamin E (mg α-TE/d) ^a^ | 10 | 10 | 10 | 10 |
| Folate (µg DFE/d) | 320 | 320 | 320 | 320 |
| ***Minerals*** |  |  |  |  |
| Calcium (mg/d) | 600 | 600 | 600 | 600 |
| Iron (mg/d) | 10.4 | 26.3 | 10.4 | 8.6 |
| Phosphorus (mg/d) | 580 | 580 | 580 | 580 |
| Zinc (mg/d) | 4.4 | 3.1 | 4.4 | 3.1 |
| Selenium (µg/d) | 30.3 | 26.3 | 30.3 | 26.3 |
| Magnesium (mg/d) ^a^ | 240 | 210 | 240 | 210 |
| Potassium (mg/d) ^a^ | 2000 | 2000 | 2000 | 2000 |

^1^Source: Philippine Dietary Reference Intake (PDRI) [24]. Values are daily Estimated Average Requirement (EAR) except ^a^ Recommended Nutrients Intake (RNI) and ^b^ Acceptable Macronutrient Distribution Range (AMDR). Abbreviations: RE (retinol equivalent), NE (niacin equivalent), α-TE (α-tocopherol equivalent), DFE (dietary folate equivalent).
