# Supplementary material for: Food sources, energy and nutrient intakes of adults: 2013 Philippines National Nutrition Survey
Source: Nutr J. 2019 Oct 10;18:59. doi: 10.1186/s12937-019-0481-z (PMC6785859; doi:10.1186/s12937-019-0481-z)
Supplement: Supplementary file 2 — Ranking of foods as major sources of energy, protein, total fat, and carbohydrates among adults (19 years and above). (DOCX 18 kb) [file 12937_2019_481_MOESM2_ESM.docx]

**Additional file 2:**  Table S2 | Ranking of foods as major sources of energy, protein, total fat, and carbohydrates among adults (19 years and above)

| **Rank** | **Energy** | |  | **Protein** | |  | **Total Fat** | |  | **Carbohydrates** | |
| --- | --- | --- | --- | --- | --- | --- | --- | --- | --- | --- | --- |
|  | **Food group** | **% of total** |  | **Food group** | **% of total** |  | **Food group** | **% of total** |  | **Food group** | **% of total** |
| **1** | Refined rice | 57.9 |  | Refined rice | 36.6 |  | Pork | 30.3 |  | Refined rice | 72.5 |
| **2** | Pork | 5.6 |  | Fish & shellfish | 21.9 |  | Fats & oils | 16.9 |  | Bread | 4.6 |
| **3** | Bread | 4.3 |  | Pork | 5.3 |  | Fish & shellfish | 7 |  | Other sweetened beverages | 2.6 |
| **4** | Fish & shellfish | 3.9 |  | Chicken | 6.8 |  | Noodles | 5.8 |  | Noodles | 2.4 |
| **5** | Noodles | 2.8 |  | Bread | 4.2 |  | Chicken | 5.5 |  | Sugar | 1.5 |
| **6** | Fats & oils | 2.4 |  | Eggs & egg dishes | 2 |  | Refined rice | 5.4 |  | Fruit, fresh | 1.5 |
| **7** | Chicken | 1.7 |  | Beef | 1.9 |  | Sausages | 4.7 |  | Soft drink, cola | 1.5 |
| **8** | Fruit, fresh | 1.2 |  | Noodles | 1.9 |  | Bread | 3.5 |  | Beans, nuts & peas | 0.7 |
| **9** | Sugar | 1.1 |  | Beans, nuts & peas | 1.8 |  | Eggs & egg dishes | 3.3 |  | Other vegetables | 0.7 |
| **10** | Soft drink, cola | 1.1 |  | Sausages | 1.6 |  | Crackers | 1.6 |  | Sweet breads | 0.7 |
| **11** | Sausages | 1 |  | Dark green leafy vegetables | 1.3 |  | Beans, nuts & peas | 1.4 |  | Cakes | 0.6 |
| **12** | Bean, nuts & peas | 0.9 |  | Other vegetables | 1 |  | Cakes | 1.4 |  | Pasta | 0.6 |
| **13** | Eggs & egg dishes | 0.8 |  | Sweet breads | 0.6 |  | Sweet breads | 1.3 |  | Crackers | 0.5 |
| **14** | Sweet breads | 0.7 |  | Milk, powdered | 0.5 |  | Beef | 1.3 |  | Dark green leafy vegetables | 0.4 |
| **15** | Other vegetables | 0.7 |  | Pasta | 0.5 |  | Milk, powdered | 1.2 |  | Deep yellow vegetables | 0.4 |
| **Total** |  | **(86.1)** |  |  | **(87.9)** |  |  | **(90.6)** |  |  | **(91.2)** |
