# Supplementary material for: Food sources, energy and nutrient intakes of adults: 2013 Philippines National Nutrition Survey
Source: Nutr J. 2019 Oct 10;18:59. doi: 10.1186/s12937-019-0481-z (PMC6785859; doi:10.1186/s12937-019-0481-z)
Supplement: Supplementary file 3 — Ranking of foods as major sources of thiamine, riboflavin, vitamin A, and vitamin C among adults (19 years and above). (DOCX 19 kb) [file 12937_2019_481_MOESM3_ESM.docx]

**Additional file 3:** Table S3 | Ranking of foods as major sources of thiamin, riboflavin, vitamin A, and vitamin C among adults (19 years and above)

| **Rank** | **Thiamine** | |  | **Riboflavin** | |  | **Vitamin A** | |  | **Vitamin C** | |
| --- | --- | --- | --- | --- | --- | --- | --- | --- | --- | --- | --- |
|  | **Food group** | **% of total** |  | **Food group** | **% of total** |  | **Food group** | **% of total** |  | **Food group** | **% of total** |
| **1** | Refined rice | 37.4 |  | Refined rice | 20.5 |  | Dark Green Leafy Vegetables | 32.3 |  | Dark Green Leafy Vegetables | 25.7 |
| **2** | Pork | 17.3 |  | Pork | 13.3 |  | Fish & Shellfish | 16.6 |  | Fruit, Fresh | 25.4 |
| **3** | Bread | 7.4 |  | Fish & Shellfish | 12.6 |  | Pork | 15.9 |  | Fruit-Based Beverages | 14.7 |
| **4** | Fish & Shellfish | 5.5 |  | Chicken | 7.2 |  | Chicken | 9.6 |  | Other Vegetables | 5.9 |
| **5** | Noodles | 4.2 |  | Dark Green Leafy Vegetables | 5.6 |  | Deep Yellow Vegetables | 6.5 |  | Other Sweetened Beverages | 5.7 |
| **6** | Other Vegetables | 3.4 |  | Eggs & Egg Dishes | 5.3 |  | Eggs & Egg Dishes | 4.4 |  | Sweet Potato | 2.4 |
| **7** | Beans, Nuts & Peas | 2.9 |  | Bread | 4.9 |  | Other Vegetables | 2 |  | Pork | 2.1 |
| **8** | Dark Green Leafy Vegetables | 2.4 |  | Milk, Powdered | 3 |  | Milk Powdered | 1.9 |  | Chicken | 2 |
| **9** | Chocolate Beverages | 2.1 |  | Other Sweetened Beverages | 2.9 |  | Bread | 1.8 |  | Deep Yellow Vegetables | 2 |
| **10** | Fruit, Fresh | 2 |  | Other Vegetables | 2.9 |  | Fruit, Fresh | 1.4 |  | Beans, Nuts & Peas | 1.1 |
| **11** | Chicken | 1.7 |  | Noodles | 1.9 |  | Chocolate Beverages | 1.1 |  | Chocolate Beverages | 0.9 |
| **12** | Pasta | 1.3 |  | Fruit, Fresh | 1.7 |  | Luncheon Meats | 1 |  | Milk Powdered | 0.6 |
| **13** | Sausages | 1 |  | Fruit-based Beverages | 1.6 |  | Beef | 1 |  | Condiments, Sauces, Herbs, Spices | 0.5 |
| **14** | Eggs & Egg Dishes | 1 |  | Beef | 1.4 |  | Cakes | 0.7 |  | Noodles | 0.3 |
| **15** | Sweet Breads | 0.8 |  | Sausages | 1.3 |  | Sausages | 0.3 |  | Cereal | 0.1 |
| **Total** |  | **(90.4)** |  |  | **(86.1)** |  |  | **(96.5)** |  |  | **(89.4)** |
