# Supplementary material for: Food sources, energy and nutrient intakes of adults: 2013 Philippines National Nutrition Survey
Source: Nutr J. 2019 Oct 10;18:59. doi: 10.1186/s12937-019-0481-z (PMC6785859; doi:10.1186/s12937-019-0481-z)
Supplement: Supplementary file 4 — Ranking of foods as major sources of folate, calcium, and iron among adults (19 years and above). (DOCX 16 kb) [file 12937_2019_481_MOESM4_ESM.docx]

**Additional file 4:** Table S4 | Ranking of foods as major sources of folate, calcium, and iron among adults (19 years and above)

| **Rank** | **Folate** | |  | **Calcium** | |  | **Iron** | |
| --- | --- | --- | --- | --- | --- | --- | --- | --- |
|  | **Food group** | **% of total** |  | **Food group** | **% of total** |  | **Food group** | **% of total** |
| **1** | Bread | 18.6 |  | Fish & Shellfish | 27.4 |  | Refined rice | 34.3 |
| **2** | Corn Grits, white | 16.4 |  | Refined rice | 23.1 |  | Fish & Shellfish | 9.8 |
| **3** | Refined rice | 11 |  | Dark Green Leafy Vegetables | 7.1 |  | Bread | 8.9 |
| **4** | Beans, Nuts & Peas | 10.8 |  | Bread | 3.2 |  | Pork | 6.9 |
| **5** | Other Vegetables | 7.8 |  | Milk Powdered | 3.1 |  | Dark Green Leafy Vegetables | 3.9 |
| **6** | Dark Green Leafy Vegetables | 6.7 |  | Chicken | 2.8 |  | Chicken | 3.2 |
| **7** | Fish & Shellfish | 3.1 |  | Other Vegetables | 2.7 |  | Sausages | 3 |
| **8** | Chicken | 3 |  | Pork | 2.6 |  | Noodles | 2.8 |
| **9** | Noodles | 2.9 |  | Fruit, Fresh | 2.5 |  | Other Vegetables | 2.4 |
| **10** | Fruit, Fresh | 2.7 |  | Condiments, Sauces, Herbs, Spices | 2.2 |  | Beans, Nuts & Peas | 2.3 |
| **11** | Crackers | 2.4 |  | Beans, Nuts & Peas | 1.7 |  | Fruit, Fresh | 2.1 |
| **12** | Eggs & Egg Dishes | 2.2 |  | Sugar | 1.5 |  | Eggs & Egg Dishes | 1.9 |
| **13** | Fruit-based Beverages | 1.5 |  | Chocolate Beverages | 1 |  | Beef | 1.6 |
| **14** | Sweet Breads | 1.3 |  | Eggs and Egg Dishes | 1 |  | Condiments, Sauces, Herbs, Spices | 1.6 |
| **15** | Pork | 1.2 |  | Cereal | 1 |  | Cereal | 1.5 |
| **Total** |  | **(91.6)** |  |  | **(82.9)** |  |  | **(86.2)** |
